# Evidence That Skeletal Muscles Modulate HDL-Cholesterol in Metabolic Healthy Young Adults

**DOI:** 10.3390/nu16081110

**Published:** 2024-04-10

**Authors:** Maria Serena Lonardo, Bruna Guida, Nunzia Cacciapuoti, Martina Chiurazzi, Daniela Pacella, Mauro Cataldi

**Affiliations:** 1 Physiology Nutrition Unit, Department of Clinical Medicine and Surgery, Federico II University of Naples, 80131 Naples, Italy; mslonardo91@gmail.com (M.S.L.); bguida@unina.it (B.G.); nunzia_cacciapuoti@libero.it (N.C.); martina.chiurazzi88@gmail.com (M.C.); 2 Department of Public Health, Federico II University of Naples, 80131 Naples, Italy; daniela.pacella@unina.it; 3 Division of Pharmacology, Department of Neuroscience, Reproductive Sciences and Dentistry, Federico II University of Naples, 80131 Naples, Italy

**Keywords:** metabolic healthy obesity, lipid profile, skeletal muscle, HDL-cholesterol

## Abstract

The aim of this study was to investigate whether skeletal muscle (SM) mass correlates with plasma lipids in metabolic healthy young adults. The study was designed as a retrospective observational monocentric study. Data on plasma lipids and SM mass of subjects attending our institution from 1999 to 2014 were analyzed. Inclusion criteria were being 18–45 years old and in apparently good health. SM mass was evaluated by bioelectrical impedance analysis (BIA) using the equation proposed by Janssen and normalized to height as skeletal muscle index (SMI: SM mass/height^2^). The association between SMI and plasma lipids levels was examined using a crude and adjusted linear regression model including age, sex, BMI and waist circumference as additional covariates. The study population consisted of 450 subjects (273 females) without metabolic syndrome (12.2% with normal body weight, 33.1% overweight, and 54.7% with obesity). SMI, total-cholesterol, LDL-cholesterol, and Triglycerides were higher, whereas HDL-cholesterol was lower in overweight and obese patients as compared with normal weight subjects. SMI was inversely associated with HDL-cholesterol in female patients with obesity but not in male patients with obesity, in normal- or over-weight subjects (*p* < 0.05). These results suggest that changes in SM mass occurring in obesity could have a role in worsening lipid profile with special reference to HDL-cholesterol.

## 1. Introduction

The physiological role of skeletal muscle (SM) is not limited to movement and posture but involves also energy metabolism control [1,2,3,4]. SMs represent, indeed, the largest insulin-sensitive tissue of the human body and account for about 20–30% of resting oxygen consumption in humans [5,6,7]. They remove about 25% of circulating glucose from the general circulation in the fasted state and 70–85% in the postprandial state [7,8,9]. Captured glucose is stored as glycogen which is then used either to support muscle contraction during exercise or as a source for maintaining glycemia during starvation. SMs also represent the main site of protein storage in the human body and about 75% of bodily proteins are stored in muscle fibers [1]. Thanks to a complex signaling network that, depending on nutrient availability, regulates the balance between protein synthesis and degradation, SM may either accumulate proteins or act as a source of circulating amino acids. In this context, SM are instrumental in the metabolic response to starvation since they provide aminoacidic substrates for hepatic gluconeogenesis [1]. In a more integrated perspective, muscle glucose is believed to be mobilized first during starvation whereas muscle protein degradation becomes more relevant at later stages when most of the glycogen stores have been used already.

While the role of skeletal muscles in glucose and protein metabolism is well established, their involvement in lipid homeostasis has been less well-determined. Nonetheless, several lines of evidence suggest that SM could have a role in controlling the levels of plasma lipids. First, SMs are a privileged site for the oxidative metabolism of lipids, which they use as a major energy source [10,11]. Specifically, during exercise, SM preferentially use circulating free fatty acids released from the adipose tissue, whereas, in resting conditions, the major source of lipids for muscle metabolism is represented by free fatty acids released from very low-density lipoprotein (VLDL) by the action of muscle lipoprotein lipase (LPL) [10,11]. Moreover, classical studies showed that, at rest, forearm skeletal muscles remove about 10% of the triglycerides (TG) infused in cannulated brachial arteries with no significant increase during exercise [12]. These data suggest that circulating TG are important to meet the energy needs of SM and that SM could contribute to their clearance from the blood. An additional, indirect argument in support of a role of SM in controlling lipid metabolism comes from the high prevalence of dyslipidemia occurring in the presence of sarcopenia, a condition characterized by the loss of muscle mass [13,14]. Likewise, sarcopenia is strongly associated with metabolic syndrome [15]. On the other hand, structural alterations in SM frequently occur in obesity and overweight since fat accumulates within the fascial envelope of muscle fibers, forming the so-called intermuscular adipose tissue (IMAT); these alterations may cause fat-induced SM dysfunction and contribute to insulin resistance [16,17]. Collectively, all of these data stand for a role of SM in regulating plasma lipid homeostasis in normal conditions and, possibly, in contributing to its alterations in overweight or obesity.

Therefore, in the present study we investigated the hypothesis that SM participate in the control of plasma lipids, by evaluating whether SM mass correlates with lipid profile in a cohort of young adults including subjects of both sexes with normal weight, overweight or obesity and no metabolic syndrome (MS). In particular, we investigated its association with low density lipoprotein-cholesterol (LDL-C), high density lipoprotein-cholesterol (HDL-C), and TG.

## 2. Materials and Methods

### 2.1. Study Design

The present study was a retrospective observational monocentric investigation performed on the medical records of patients attending the outpatient clinic of the Physiology Nutrition Unit of the Federico II University of Naples from 1999 to 2014 for a dietary consultation and correction of eating habits. The study was conducted according to the guidelines of the Declaration of Helsinki and approved by the Federico II Ethics Committee (protocol code 481/21, date of approval 26 January 2022) and all of the subjects enrolled gave informed consent before the beginning of the study. Inclusion criteria were age between 18 and 45 years, absence of MS according to National Cholesterol Education Program (NCEP) modified Adult Treatment Panel III (ATP III) criteria [18], no concurrent disease, and no drug therapy at the time of the consultation. Exclusion criteria were age less than 18 or higher than 45, pregnancy or lactation, menopausal status, concomitant diseases, drug therapy, and pathological hydration status detected by Bioelectrical Impedance Vector Analysis (BIVA). We also excluded patients with MS diagnosed according to NCEP modified ATP III criteria (i.e., the presence of at least t 3 of the following conditions: abdominal obesity (waist circumference: >102 cm in men and >88 cm in women); fasting serum triglycerides ≥1.7 mmol/L (≥150 mg/dL) or ongoing drug treatment for hypertriglyceridemia; serum HDL cholesterol concentrations <1.0 mmol/L (<40 mg/dL) in men, or <1.3 mmol/L (<50 mg/dL) in women, or specific lipid lowering drug therapy; systolic blood pressure (SBP) ≥130 mmHg; diastolic blood pressure ≥85 mmHg or current antihypertensive drug treatment; fasting blood glucose ≥6.1 mmol/L (≥100 mg/dL) or specific antidiabetic drug therapy or previous diagnosis of type 2 diabetes mellitus [18]. No restriction was set on the Body Mass Index (BMI). Patients with a story of dietary interventions, strenuous physical exercise programs for weight reduction during the six months preceding the consultation or bariatric surgery were excluded as well. Women of reproductive age were included in the study irrespective of the phase of the menstrual cycle since this information was not systematically collected at the time of consultation. From the medical records of the patients matching the inclusion criteria, anthropometric, bioimpedance, and biochemical data that are routinely recorded during patient appointments at our outpatient clinic were collected as detailed in the following paragraphs.

### 2.2. Anthropometric, Body Composition and Laboratory Parameters

Patients’ anthropometric evaluation included the measurement of height, weight, waist circumference, and BMI. Height and weight were detected with a calibrated stadiometer and scale, while waist circumference was measured at the midpoint between the lower border of the ribcage and the iliac crest by using a flexible inch tape directly applied to the skin. BMI was defined as the weight in kilograms divided by the square of the height in meters. According to the World Health Organization (WHO) cutoff values [19], patients were classified as normal weight (NW), overweight (OW) and obese (OB) if their BMI was in the range 18.5–24.9, 25–29.9, and ≥ 30 kg/m^2^, respectively. The accuracy of this measure was about 0.1 cm.

Body composition was assessed with bioelectrical impedance analysis (BIA) using a tetrapolar 50-kHz bioelectrical impedance analyzer (BIA101RJL, Akern Bioresearch, Firenze, Italy) [20,21]. Total body water (TBW), fat mass (FM), and fat free mass (FFM) were extrapolated from the values of Reactance (Xc) and Resistance (Rs) obtained from BIA by using specific prediction equations. Skeletal muscle (SM) mass (in kg) was calculated from BIA-measured resistance using the equation proposed by Janssen [22]:

SM = [(height^2^/Rs·0.401) + (sex·3.825) + (−0.071·age)] + 5.102

where sex = 1 in men and 0 in women. SM was normalized to height and expressed as SM/height^2^ (SMI). FM was normalized to height squared (FM/height^2^) and expressed as Fat Mass Index (FMI). Bioelectrical Vector Analysis (BIVA) was performed offline with the BIVA software Biavector^®^ version 2.1.5 (Akern Bioresearch, Firenze, Italy). This software identifies the position of individual bioelectrical vectors on the RXc point graph concerning tolerance ellipses that represent the 50%, 75%, and 95% of the values observed in a reference population. The length of the vector is an index of the hydration status since a vector shorter than normal (low resistance) is associated with fluid overload and a vector longer than normal (high resistance) with dehydration [23].

The following biochemical parameters were measured using standard techniques in blood samples collected in the morning from all patients after overnight fasting: total, HDL and LDL cholesterol, triglycerides, glycemia (Gly), Glutamic-Oxalacetic Transaminase (GOT), Glutamic Pyruvic Transaminase (GPT), serum albumin and total serum protein.

### 2.3. Statistical Analysis

Data are reported as mean and standard deviation for continuous variables and as absolute frequencies and percentages for categorical variables. The normality of the distributions was assessed using the Shapiro-Wilk test. After conducting sensitivity analysis, we removed from the study cohort the outliers that were identified as those cases whose values of either HDL-cholesterol, LDL-cholesterol, triglycerides or SMI were lower than Q1 − (1.5 × IRQ) or higher than Q3 + (1.5 × IRQ), where IRQ is the interquartile range, and Q1 and Q3, the first and the third quartile values (original cohort 464 patients, 450 after outlier removal). The difference between means for two group comparisons was performed with the student’s *t*-test for independent samples or with the Mann Whitney U test as appropriate. Comparison of means among three or more groups was assessed with ANOVA or with Kruskal-Wallis as appropriate. To assess whether plasma lipids differ in subjects with different SMI, we stratified NW, OW and OB subjects in SMI tertiles by subdividing the patients in three groups each containing one third of the population with the first tertile corresponding to the lowest SMI values and the third tertile corresponding to the highest SMI values (SMI cutoff values in kg/m^2^: 9.38 and 10.13 for NW, 9.58 and 11.36 for OW, and 11.05 and 12.43 for OB). Then, we compared the levels of HDL-cholesterol, LDL-cholesterol and triglycerides among the different tertiles. The associations between SMI and all lipid plasma concentrations were examined using a crude and adjusted linear regression model including age, sex, BMI and waist circumference as additional covariates. For all analyses, a *p* < 0.05 was considered significant. Analyses were conducted using R statistical software version 4.3.0.3.

## 3. Results

### 3.1. Study Population

After outlier removal, the study population consisted of 450 subjects (273 females; 60.66%) with a median age of 30 [24–35.8] years: 55 (12.22%) of them were a normal weight, 149 (33.1%) overweight, and the remaining 246 (54.7%) were affected by obesity. According to inclusion criteria, the enrolled subjects were all in apparent good health, none of them met the criteria for the metabolic syndrome or was taking any drug therapy at the time of the medical consultation. Table 1 summarizes the anthropometric, body composition, and laboratory parameters of the enrolled subjects stratified by BMI classes. Significant differences in body composition were observed across the different BMI classes. As expected, not only FMI but also SMI values were higher in participants with overweight as compared with normal weight subjects and in patients with obesity as compared with both normal and overweight subjects. Therefore, not only fat but also muscle mass increased as body weight increased. Furthermore, BIVA showed that, when BMI increased, vector length decreased, and phase angle proportionally increased. These results suggest that TBW increased proportionally with extracellular water (ECW) due to the increase in muscle and adipose cell number with no change in tissue hydration. In addition, lipid profile was significantly worse in obese than in normal or overweight subjects, with significantly higher LDL cholesterol, and triglycerides and lower HDL-cholesterol (Table 1).

### 3.2. Association between SMI and All Plasma Lipid Concentrations

**Figure 1:**
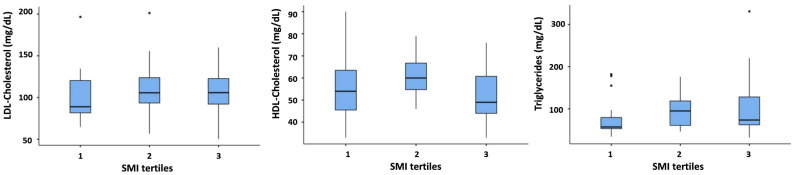
Comparison of LDL-cholesterol, HDL-cholesterol, and triglycerides among different SMI tertiles in normal weight subjects. In each box plot, the thick line in the middle represents the median of the values considered, the box itself extends from the 25th (on the bottom of the box) to the 75th percentile value (on the top of the box), the upper whisker goes to the 75th percentile + (1.5 × IQR) and the lower whisker to the 25th percentile − (1.5 × IQR) whereas the dots indicate the outlier values.

**Figure 2:**
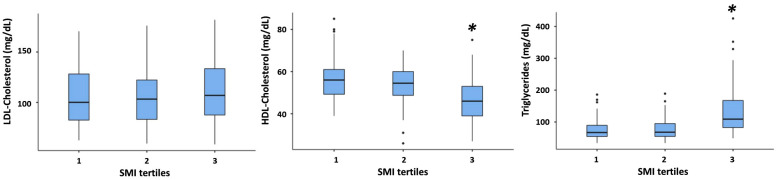
Comparison of LDL-cholesterol, HDL-cholesterol, and triglycerides among different SMI tertiles in overweight subjects. * = *p* < 0.001 vs. 1st and 2nd tertile. See the legend to Figure 1 for an explanation of the box plot.

**Figure 3:**
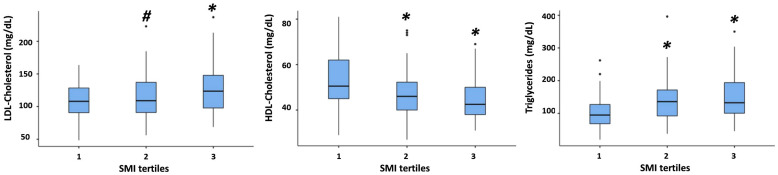
Comparison of LDL-cholesterol, HDL-cholesterol, triglycerides, and TG/HDL ratio among different SMI tertiles in patients with obesity. * = *p* < 0.001 vs. 1st tertile; # = *p* < 0.001 vs. 3rd tertile. See the legend to Figure 1 for an explanation of the box plot.

Since in patients with obesity the increase in SMI occurs in parallel with the worsening of lipid profile, we hypothesized that these two phenomena could be somehow interrelated the one with the other. To investigate this hypothesis, we compared the plasma concentrations of HDL-cholesterol, LDL-cholesterol and triglycerides in patients belonging to the three different SMI tertiles (see Supplementary Tables S1–S3 for numeric data). The results of these comparisons in normal subjects, overweight subjects and patients with obesity are reported, respectively, in Figure 1, Figure 2 and Figure 3. No difference was observed among SMI tertiles in any of the plasma lipid parameters in normal subjects (Figure 1).

By contrast, in overweight subjects, TG were higher, and HDL-cholesterol was lower in the third SMI tertiles as compared with the other two (Figure 2).

Finally, in patients with obesity, TG were higher, and HDL-cholesterol was lower both in the second and in the third SMI tertile as compared with the first one, whereas LDL-cholesterol was higher in the in the third tertile as compared with the other two (Figure 3).

**Figure 4:**
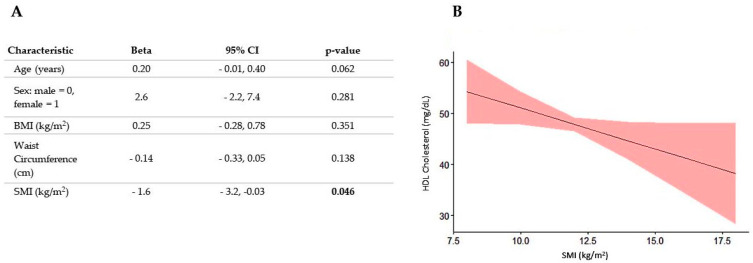
Regression analysis of the association between SMI and HDL-cholesterol in subjects affected with obesity. Numeric parameters for SMI and covariates are reported in panel (**A**) whereas the regression line with confidence intervals is reported in panel (**B**). The values in bold indicate statistically significant values.

To further investigate whether skeletal muscle could influence plasma lipid profile, we performed a regression analysis with the specific aim of excluding the role of covariates known to affect plasma which increase in parallel with BMI exactly as SMI does. More specifically, we introduced as covariates in our analysis age, sex, BMI and waist circumference. The results of this analysis, reported in Figure 4, showed that after correcting for covariates, HDL-cholesterol was significantly associated with SMI but only in patients with obesity and not in normal or overweight subjects. Quite unexpectedly, there was an inverse relationship between these two variables since HDL-cholesterol decreased as SMI increased.

By contrast, SMI was not correlated either with total cholesterol, LDL-cholesterol, or triglycerides in any of the examined subgroups (normal weight, overweight and obese) (Supplementary Table S4).

**Figure 5:**
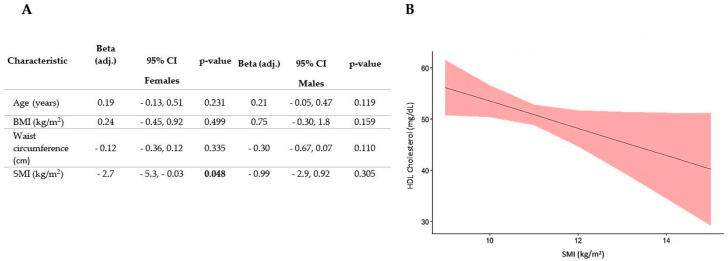
Regression analysis of the association between SMI and HDL-cholesterol in female and male subjects with obesity. Panel (**A**) reports the numeric parameters of the regression for both sexes whereas panel (**B**) shows the regression line with confidence intervals for the female subjects only. The values in bold indicate statistically significant values.

To investigate whether there was any sex-related effect in the relationship between SMI and plasma HDL in patients with obesity we performed regression analysis of male and female data separately. The results of this analysis showed that SMI was related to HDL only in females (Figure 5).

## 4. Discussion

In the present study we evaluated whether the increase in SM mass that occurs in patients with obesity is related with the worsening of lipid profile also occurring in this condition. In support of this hypothesis, the results that we obtained showed that SMI is an independent predictor of low HDL-cholesterol in female but not in male patients with obesity. Importantly, our study population was composed of young adults with no diabetes, metabolic syndrome or known cardiovascular diseases suggesting that the association between SM and lipid metabolism could occur early in the natural history of obesity. 

These findings may appear counter-intuitive since body fitness and SM mass are known to be associated with high insulin sensitivity and low cardiovascular risk. However, as reviewed by Lagacé et al. [24], evidence has been accumulating that a high FFM (presumably mainly determined by a higher SM mass) may be deleterious to metabolic health at least in certain circumstances. For instance, Brochu et al. [25] showed that in a cohort of obese postmenopausal women, the metabolic profile was worse in those with a higher lean mass. In addition, data from the Amsterdam Growth and Health Study showed that HDL-C concentrations were inversely related to FFM [26] and Pietrobelli et al. [27] reported that HDL-C concentration negatively correlates with lean body mass and skeletal muscle. More recently, results from a subset of metabolic healthy adults, participating in the Fels Longitudinal Study, showed that, during the 26 years of follow-up of the study, increases in fat free mass index (FFMI), as well as in FMI, were associated with decreases in HDL-C and increases in TG [28]. Comparable results were reported also by Duran et al. [29] who showed an inverse association between muscle mass and HDL-cholesterol concentration in a cohort of 6288 children and adolescents, suggesting a counterintuitive, inverse association of muscle mass and HDL-cholesterol concentration [29]. 

The mechanism responsible for the sex-dependent inverse correlation between SMI and HDL-cholesterol is unclear. The evidence that, in our study population, SMI was inversely associated with HDL-cholesterol only in patients with obesity but not in normal or overweight subjects suggests that muscle alterations specifically occurring in obesity could be involved. It is tempting to speculate that, in non-obese subjects, SMs contribute to keeping high the circulating levels of HDL and that this mechanism is impaired in obesity. As a matter of fact, metabolic muscle dysfunction may occur in obesity. In fact, fatty infiltration with IMAT deposition and intracellular accumulation of lipids inside myocytes is commonly observed in obesity [30,31]. These alterations reduce muscle oxidative capacity while at the same time increasing anaerobic and glycolytic capacities and are responsible for metabolic dysfunction since they cause microinflammation, insulin resistance, and a decrease in the expression/activity of muscle LPL [32,33,34,35,36]. While the primary physiological role of muscle LPL is to remove free fatty acids from VLDL and promote VLDL conversion into Triglyceride-rich lipoproteins (TRL), this enzyme also controls HDL plasma concentrations. As a matter of fact, in the process of the LPL-mediate hydrolysis of TRL, surface free cholesterol, phospholipids and apolipoproteins are transferred to HDL particles hence contributing to increase plasma HDL [37]. In addition, Cholesteryl ester transfer protein (CETP) may transfer lipids from LPL-generated TRL to HDL further increasing their concentration [37]. In this scenario, a decrease in muscle LPL activity caused by fatty infiltration and the consequent reduction of insulin sensitivity is expected to cause a reduction in plasma HDL. Several arguments support the link between muscle fatty infiltration, and the reduction of insulin sensitivity and LPL activity. It has been observed, indeed, that LPL activity in skeletal muscle negatively correlates with plasma insulin levels and it has been suggested that it could be lowered by insulin resistance [38]. Importantly, evidence has been reported that fatty infiltration of skeletal muscles on computer tomography (CT) scan correlates with insulin resistance and its metabolic consequences. For instance, in a group of obese but nondiabetic adults from South Korea, Kim et al. [39] observed that the area of low attenuation in mid-thigh muscle (which corresponds to fat-infiltrated muscles) positively correlated with Homeostasis Model Assessment (HOMA) index, serum insulin, and free fatty acid concentrations. Moreover, LPL activity in skeletal muscle negatively correlates with plasma insulin levels and could be lowered by insulin resistance [37]. A recent lipidomic study showed that higher VLDL and lower HDL concentrations were associated with higher muscle area, higher IMAT area, and lower IMAT density (an index of muscle fatty infiltration) [31]. Therefore, low HDL-cholesterol in patients living with obesity and with higher fat mass could simply be one of the consequences of the decrease in the insulin sensitivity of skeletal muscles caused by muscle fat infiltration. 

Insulin sensitivity and LPL expression are also linked to another factor which can be altered in obesity: the prevalence of the different types of fibers in skeletal muscle. Specifically, in patients with obesity, as compared with normal weight individuals the prevalence of type IIb fibers is higher and that of type I/SO and type IIa fibers is lower [40,41]. Type I and IIa oxidative muscle fibers contribute to the clearance of LDL and VLDL from the blood to a greater extent than type IIb glycolytic fibers due to a higher expression of LPL and fatty acid binding and transport proteins [16,41,42,43]. Tikkanen et al. [44] showed that, indeed, a higher prevalence of oxidative fibers in SM was associated with higher HDL-C and lower TG values possibly due to the higher expression in these fibers of LPL which degrades TGs and contributes to HDL-C production [44]. Intriguingly, it has been recently suggested that muscle mass fiber composition could be a major factor influencing cardiovascular risk [16]. Moreover, fiber composition of SM could be a determinant of the risk of developing obesity [43] with higher risk in individuals with a high prevalence of type IIx/IIb fibers and a lower risk in those with a high prevalence of type I muscle fibers [16]. The different representation of Type I/IIa and type IIb muscle fibers in males and in females could help explaining the sex dimorphism that we observed in the correlation between HDL and SMI. In fact, type I muscle fibers are more represented in female than in male skeletal muscle making the role of muscle fibers in lipid metabolism potentially more relevant in females than in males [45,46] and the consequence of obesity-induced muscle metabolic dysfunction more severe in females than in males. It should also be considered that the higher representation of type I fibers in female skeletal muscles increases their ability to accumulate intracellular lipids and, potentially, to undergo lipid-mediated cell damage [47].

The relationship between HDL-cholesterol and SM could be bidirectional with muscle metabolic dysfunction contributing to decrease HDL-cholesterol and low HDL-cholesterol playing a role in causing muscle metabolic dysfunction. In fact, Marron et al. (2021) [31] showed that IMAT accumulation could be facilitated by an inefficient reverse cholesterol transport. Moreover, it has been demonstrated that, in mice, HDL-cholesterol is required to preserve mitochondrial function and oxidative metabolism in SM [48]. 

Our study has several limitations. First, we only evaluated muscle mass with BIA and therefore we do not have any morphological data such as those provided by CT scan or magnetic resonance imaging (MRI) that could give relevant information on muscle quality with special reference to fatty infiltration. Second, our study had a cross-sectional design and, therefore, we cannot establish how stable its findings were over time. In addition, in the absence of long-term follow-up, we can only speculate on the possible implications of our findings on metabolic and SM health. We have no data about the insulin profile of our study population and, actually, most of the subjects turned out to be overweight/obese despite having no diagnosed pathology. Finally, we did not ask our female patients to come to the observation at a specific time of their menstrual cycle and, therefore, the data we obtained were presumably scattered across different menstrual phases. This could be relevant considering that significant changes in plasma lipid have been observed during the menstrual cycle. In particular, it has been shown that total cholesterol, triglycerides, and LDL cholesterol reach maximum levels during the follicular phase and then decrease during the luteal phase, while plasma levels of HDL cholesterol are higher at the time of ovulation, and quite stable during the other phases of the menstrual cycle [49].

## 5. Conclusions

This study showed that SM mass is inversely related to HDL-cholesterol in metabolic healthy female young subjects with obesity. Our data do not allow any definite conclusion on whether muscle dysfunction has a causative role in lowering HDL-cholesterol or, instead, HDL-cholesterol alters SM mass and function. Further studies will be necessary to establish whether, due to its association with plasma lipids, SMI could represent new predictor of cardiovascular risk to be added to the classical cardiovascular risk predictors related to body composition (which for decades have been all related to fat mass and especially visceral fat accumulation) [50,51]. 

## Figures and Tables

**Table 1:** Anthropometric, body composition and laboratory characteristics of the study population.

	NW N = 55	OW N = 149	OB N = 246	*p*-Value
Anthropometric Parameters				
Age (years)	28 ± 7 [26, 30] ^b^	29 ± 7 [28, 30]	31 ± 7 [30, 31]	0.031 *
Weight (kg)	63 ± 8 [61, 65] ^a,b^	77 ± 10 [76, 79] ^c^	98 ± 15 [96, 100]	<0.001 **
Height (cm)	165 ± 9 [162, 167]	166 ± 9 [165, 168] ^c^	166 ± 10 [165, 168]	0.398
BMI (kg/m^2^)	23.1 ± 1.4 [23, 24] ^a,b^	27.8 ± 1.5 [28, 28] ^c^	35.3 ± 4.1 [35, 36]	<0.001 **
Waist Circumference (cm)	80 ± 6 [79, 82] ^a,b^	92 ± 8 [91, 94] ^c^	109 ± 11 [107, 110]	<0.001 **
Body composition				
R (Ω)	585 ± 68 [567, 604] ^a,b^	543 ± 75 [530, 555] ^c^	474 ± 60 [466, 481]	<0.001 **
Xc (Ω)	68 ± 8 [66, 70] ^a,b^	65 ± 8 [64, 66] ^c^	60 ± 8 [59, 61]	<0.001 **
Φ (degrees)	6.67 ± 0.80 [6.5, 6.9] ^b^	6.90 ± 0.87 [6.8, 7.0] ^c^	7.25 ± 0.91 [7.1, 7.4]	<0.001 **
Vector Length (Ω/m)	361 ± 53 [346, 375] ^a,b^	330 ± 55 [321, 339] ^c^	289 ± 46 [283, 295]	<0.001 **
FMI (kg/m^2^)	10 ± 3 [9.2, 11] ^a,b^	15 ±3 [14, 15] ^c^	22 ± 6 [22, 23]	<0.001 **
SMI (kg/m^2^)	9.88 ± 1.15 [9.6, 10] ^a,b^	10.59 ±1.49 [10, 11] ^c^	11.83 ± 1.45 [12, 12]	<0.001 **
Laboratory parameters				
Plasma Glucose (mg/dL)	84 ± 8 [82, 86] ^b^	87 ± 10 [85, 89] ^c^	90 ± 11 [88, 91]	<0.001 **
Total Cholesterol (mg/dL)	181 ± 39 [170, 191]	179 ± 33 [174, 184] ^c^	190 ± 36 [185, 195]	0.007 **
HDL-Cholesterol (mg/dL)	56 ± 13 [52, 59] ^b^	52 ± 11 [51, 54] ^c^	48 ± 11 [47, 49]	<0.001 **
LDL-Cholesterol (mg/dL)	107 ± 31 [98, 115] ^b^	106 ± 28 [102, 111] ^c^	116 ± 32 [112, 120]	0.005 **
Triglycerides (mg/dL)	92 ± 56 [77, 107] ^b^	100 ± 66 [90, 111] ^c^	130 ± 62 [122, 138]	<0.001 **

NW = Normal weight; OW = Overweight; Ob = Obese. Data reported as mean ± s.d. with [95% confidence intervals]. *p*-value computed with ANOVA or Kruskal Wallis’s as appropriate. * = *p* < 0.05; ** = *p* < 0.01; ^a^ *p* < 0.05 vs. Overweight; ^b^ *p* < 0.05 vs. Obese; ^c^ *p* < 0.05 vs. Obese.

## Data Availability

The data are stored in a database at the Department of Clinical Medicine and Surgery, Nutrition Physiology Unit, University Federico II of Naples, Naples 80131, Italy. It is available upon request to be made to Bruna Guida.

## Supplementary Materials

The following supporting information can be downloaded at: https://www.mdpi.com/article/10.3390/nu16081110/s1, Table S1: Tertiles normal weight; Table S2: Tertiles over-weight; Table S3: Tertiles obese; Table S4: Regresssion data.

## Abbreviations

ATP III	Adult Treatment Panel III
BIA	Bioelectrical Impedance Analysis
BIVA	Bioelectrical Vector Analysis
BMI	Body Mass Index
CETP	Cholesteryl ester transfer protein
CT	Computed Tomography
ECW	Extracellular Water
FFM	Fat Free Mass
FFMI	Fat Free Mass Index
FM	Fat Mass
FMI	Fat Mass Index
Gly	Glycemia
GOT	Glutamic-Oxalacetic Transaminase
GPT	Glutamic Pyruvic Transaminase
HDL-C	High-Density Lipoprotein-Cholesterol
HOMA index	Homeostasis Model Assessment index
IMAT	Intermuscular Adipose Tissue
IRQ	Interquartile range
LDL-C	Low Density Lipoprotein-Cholesterol
LPL	Lipoprotein Lipase
MRI	Magnetic Resonance Imaging
MS	Metabolic Syndrome
NCEP	National Cholesterol Education Program
NW	Normal weight
Ob	Obese
OW	Overweight
Q1	first quartile
Q3	third quartile
Rs	Resistance
SM	Skeletal Muscle
SMI	Skeletal Muscle Index
TBW	Total body water
TBW	Total Body Water
TG	Triglycerides
TRL	Triglyceride-rich lipoproteins
VLDL	Very Low-Density Lipoproteins
WHO	World Health Organization
Xc	Reactance
