# Supplementary material for: Evidence That Skeletal Muscles Modulate HDL-Cholesterol in Metabolic Healthy Young Adults
Source: Nutrients. 2024 Apr 10;16(8):1110. doi: 10.3390/nu16081110 (PMC11054046; doi:10.3390/nu16081110)
Supplement: Supplementary file 1 [file nutrients-16-01110-s001.zip › Table S1. Tertiles normal weight.pdf]

PATIENT NUMBER	SMI	SMI tertiles	HDL-Cholesterol (mg/dL)	LDL-Cholesterol (mg/dL)	Triglycerides (mg/dL)
1	8,16	1	57,00	134,60	97,00
2	8,21	1	40,00	81,20	89,00
3	8,26	1	65,00	132,00	155,00
4	8,33	1	52,00	84,40	48,00
5	8,46	1	48,00	102,60	182,00
6	8,63	1	44,00	65,00	65,00
7	8,64	1	33,00	98,20	44,00
8	8,69	1	40,00	65,60	52,00
9	8,75	1	66,00	87,20	54,00
10	8,87	1	57,00	131,60	57,00
11	8,89	1	51,00	79,40	178,00
12	8,93	1	39,00	109,20	34,00
13	8,98	1	71,00	91,60	57,00
14	8,99	1	80,00	82,20	39,00
15	9,05	1	62,00	86,40	68,00
16	9,07	1	47,00	74,60	62,00
17	9,28	1	62,00	196,60	57,00
18	9,37	1	90,00	133,60	57,00
19	9,38	1	54,00	89,00	70,00
20	9,41	2	47,00	105,20	139,00
21	9,46	2	51,00	58,80	46,00
22	9,48	2	51,00	102,80	61,00
23	9,49	2	69,00	153,60	112,00
24	9,52	2	64,00	128,80	106,00
25	9,54	2	61,00	56,80	56,00
26	9,54	2	59,00	93,80	176,00
27	9,55	2	66,00	201,20	104,00
28	9,62	2	79,00	94,00	60,00
29	9,63	2	46,00	108,20	79,00
30	9,64	2	70,00	92,80	71,00
31	9,70	2	57,00	122,20	54,00
32	9,78	2	59,00	155,80	106,00
33	9,96	2	79,00	106,20	139,00
34	9,99	2	56,00	92,40	158,00
35	10,05	2	66,00	116,80	86,00
36	10,13	3	65,00	138,20	89,00
37	10,13	3	34,00	97,60	32,00
38	10,13	3	67,00	80,80	66,00
39	10,22	3	49,00	98,40	63,00
40	10,28	3	50,00	50,40	93,00
41	10,48	3	38,00	128,80	126,00
42	10,49	3	76,00	123,20	49,00
43	10,56	3	50,00	82,00	65,00
44	10,63	3	42,00	112,80	51,00
45	10,87	3	49,00	94,20	44,00
46	11,09	3	66,00	101,20	59,00
47	11,32	3	33,00	151,80	331,00
48	11,34	3	47,00	92,20	64,00
49	11,59	3	46,00	92,20	174,00
50	11,64	3	50,00	86,00	135,00
51	11,76	3	63,00	116,20	64,00
52	11,84	3	44,00	122,60	87,00
53	11,91	3	44,00	110,60	167,00
54	12,46	3	60,00	160,00	220,00
55	13,28	3	47,00	110,80	81,00
