# Supplementary material for: Evidence That Skeletal Muscles Modulate HDL-Cholesterol in Metabolic Healthy Young Adults
Source: Nutrients. 2024 Apr 10;16(8):1110. doi: 10.3390/nu16081110 (PMC11054046; doi:10.3390/nu16081110)
Supplement: Supplementary file 1 [file nutrients-16-01110-s001.zip › Table S2. Tertiles over-weight.pdf]

PATIENT NUMBER	SMI	SMI tertiles	HDL-Colesterol (mg/dL)	LDL-Colesterolo (mg/dL)	Triglycerides (mg/dL)
1	7,58	1	56,00	139,60	57,00
2	8,20	1	49,00	131,00	170,00
3	8,28	1	58,00	82,20	54,00
4	8,40	1	54,00	68,00	40,00
5	8,49	1	60,00	101,00	70,00
6	8,49	1	56,00	72,00	40,00
7	8,55	1	56,00	82,40	43,00
8	8,58	1	50,00	63,74	51,30
9	8,61	1	54,00	126,00	110,00
10	8,61	1	48,00	98,00	65,00
11	8,76	1	60,00	101,40	63,00
12	8,78	1	61,00	62,40	38,00
13	8,88	1	71,00	170,60	162,00
14	8,89	1	71,00	154,40	58,00
15	8,89	1	58,00	97,00	70,00
16	8,89	1	68,00	68,80	126,00
17	8,96	1	40,00	106,60	47,00
18	8,96	1	61,00	152,20	69,00
19	8,97	1	56,00	104,40	88,00
20	8,98	1	47,00	143,00	80,00
21	8,99	1	56,00	97,40	68,00
22	9,00	1	56,00	132,80	61,00
23	9,03	1	54,00	136,80	66,00
24	9,05	1	52,00	81,40	43,00
25	9,05	1	41,00	94,80	41,00
26	9,15	1	47,00	99,40	88,00
27	9,15	1	59,00	133,00	90,00
28	9,18	1	78,00	78,20	104,00
29	9,22	1	47,00	83,20	124,00
30	9,24	1	50,00	106,80	66,00
31	9,26	1	67,00	102,60	37,00
32	9,27	1	55,00	91,80	186,00
33	9,28	1	46,00	82,00	60,00
34	9,33	1	51,00	83,80	76,00
35	9,36	1	63,00	100,80	41,00
36	9,36	1	45,00	96,80	101,00
37	9,37	1	40,00	131,60	112,00
38	9,38	1	49,00	130,40	88,00
39	9,39	1	70,00	118,40	68,00
40	9,42	1	57,00	89,60	142,00
41	9,48	1	50,00	74,40	58,00
42	9,48	1	68,00	91,40	73,00
43	9,50	1	80,00	129,00	110,00
44	9,50	1	85,00	113,40	58,00
45	9,51	1	57,00	71,00	65,00
46	9,54	1	63,00	113,80	56,00
47	9,55	1	39,00	82,60	82,00
48	9,55	1	49,00	144,20	104,00
49	9,56	1	50,00	103,60	37,00
50	9,58	1	79,00	71,20	34,00
51	9,59	2	62,00	82,60	67,00
52	9,61	2	68,00	106,80	36,00
53	9,62	2	56,00	114,20	89,00
54	9,64	2	70,00	80,20	49,00
55	9,66	2	64,00	64,20	64,00
56	9,71	2	48,00	84,00	95,00
57	9,72	2	67,00	80,80	46,00
58	9,74	2	49,00	127,80	111,00
59	9,75	2	39,00	83,20	119,00
60	9,80	2	52,00	85,40	88,00
61	9,81	2	62,00	105,80	101,00
62	9,81	2	55,00	98,40	153,00
63	9,82	2	58,00	84,20	49,00
64	9,89	2	59,00	83,20	109,00
65	9,97	2	53,00	70,00	90,00
66	9,98	2	47,00	59,20	64,00
67	9,98	2	50,00	123,00	75,00
68	10,01	2	54,00	80,20	94,00
69	10,03	2	54,00	98,40	68,00
70	10,05	2	58,00	144,80	56,00

71	10,09	2	60,00	67,00	60,00
72	10,12	2	57,00	85,80	41,00
73	10,24	2	55,00	106,20	54,00
74	10,27	2	49,00	106,40	58,00
75	10,36	2	31,00	128,40	103,00
76	10,37	2	55,00	121,80	51,00
77	10,39	2	56,00	88,80	61,00
78	10,39	2	49,00	127,40	113,00
79	10,51	2	64,00	135,60	72,00
80	10,61	2	60,00	102,80	51,00
81	10,62	2	53,00	105,00	95,00
82	10,63	2	47,00	100,40	68,00
83	10,77	2	50,00	138,00	75,00
84	10,77	2	60,00	95,80	56,00
85	10,86	2	63,00	64,40	83,00
86	10,89	2	68,00	107,00	165,00
87	10,95	2	41,00	68,80	46,00
88	10,97	2	49,00	171,00	90,00
89	11,00	2	65,00	122,00	65,00
90	11,02	2	48,00	117,20	69,00
91	11,15	2	26,00	176,20	189,00
92	11,17	2	43,00	131,00	140,00
93	11,21	2	37,00	103,80	51,00
94	11,24	2	51,00	90,20	34,00
95	11,25	2	48,00	144,40	143,00
96	11,26	2	46,00	125,80	56,00
97	11,31	2	58,00	115,60	52,00
98	11,33	2	65,00	76,00	55,00
99	11,36	3	56,00	125,80	81,00
100	11,36	3	49,00	64,60	91,00
101	11,37	3	52,00	101,40	103,00
102	11,40	3	48,00	132,20	49,00
103	11,44	3	63,00	112,00	425,00
104	11,45	3	43,00	116,20	79,00
105	11,45	3	54,00	98,00	50,00
106	11,46	3	50,00	121,60	157,00
107	11,48	3	52,00	97,20	154,00
108	11,62	3	54,00	143,60	162,00
109	11,66	3	59,00	82,20	94,00
110	11,70	3	57,00	114,40	68,00
111	11,71	3	58,00	92,60	152,00
112	11,73	3	54,00	163,60	92,00
113	11,81	3	42,00	98,20	109,00
114	11,84	3	52,00	148,60	352,00
115	11,90	3	46,00	130,80	106,00
116	12,01	3	50,00	164,00	70,00
117	12,02	3	37,00	101,40	213,00
118	12,08	3	35,00	85,80	101,00
119	12,08	3	38,00	75,80	106,00
120	12,11	3	39,00	76,40	88,00
121	12,11	3	47,00	122,60	132,00
122	12,11	3	32,00	104,80	241,00
123	12,13	3	55,00	98,20	294,00
124	12,17	3	35,00	80,80	241,00
125	12,20	3	37,00	144,80	71,00
126	12,26	3	43,00	89,20	54,00
127	12,29	3	27,00	100,40	168,00
128	12,36	3	68,00	58,40	68,00
129	12,38	3	31,00	65,60	287,00
130	12,42	3	45,00	153,40	133,00
131	12,50	3	42,00	117,00	85,00
132	12,51	3	38,00	82,60	167,00
133	12,52	3	40,00	84,40	158,00
134	12,52	3	46,00	108,20	284,00
135	12,53	3	46,00	155,20	329,00
136	12,54	3	60,00	107,00	60,00
137	12,56	3	62,00	97,00	60,00
138	12,69	3	45,00	91,00	145,00
139	12,77	3	36,00	122,00	60,00
140	12,79	3	46,00	182,00	200,00
141	12,79	3	32,00	82,20	84,00

142	12,82	3	50,00	137,80	121,00
143	13,34	3	75,00	62,60	62,00
144	13,42	3	48,00	134,80	116,00
145	13,45	3	42,00	137,40	98,00
146	13,64	3	50,00	166,60	122,00
147	13,72	3	33,00	85,00	205,00
148	14,38	3	41,00	150,00	260,00
149	15,20	3	39,00	127,40	103,00
