# Supplementary material for: Evidence That Skeletal Muscles Modulate HDL-Cholesterol in Metabolic Healthy Young Adults
Source: Nutrients. 2024 Apr 10;16(8):1110. doi: 10.3390/nu16081110 (PMC11054046; doi:10.3390/nu16081110)
Supplement: Supplementary file 1 [file nutrients-16-01110-s001.zip › Table S3. Tertiles obese.pdf]

PATIENT NUMBER	SMI	SMI tertiles	HDL-Colesterol (mg/dL)	LDL-Cholesterol (mg/dL)	Triglycerides (mg/dL)
1	9,05	1	46,74	91,26	70,00
2	9,16	1	45,00	86,80	131,00
3	9,27	1	72,00	99,00	60,00
4	9,32	1	54,00	104,40	78,00
5	9,39	1	33,00	127,40	98,00
6	9,40	1	52,00	48,40	128,00
7	9,51	1	54,00	90,60	67,00
8	9,58	1	41,00	128,80	151,00
9	9,62	1	47,00	122,60	72,00
10	9,63	1	71,00	109,80	136,00
11	9,68	1	41,00	150,20	104,00
12	9,76	1	58,00	120,60	92,00
13	9,76	1	60,00	81,00	20,00
14	9,78	1	65,00	93,60	117,00
15	9,78	1	45,00	107,80	51,00
16	9,79	1	72,00	115,60	122,00
17	9,84	1	48,00	84,40	73,00
18	9,90	1	41,00	74,20	49,00
19	9,96	1	46,00	108,80	146,00
20	9,96	1	50,00	66,40	68,00
21	10,00	1	64,00	96,40	73,00
22	10,00	1	60,00	145,80	91,00
23	10,01	1	52,00	118,80	56,00
24	10,02	1	64,00	120,00	80,00
25	10,02	1	50,00	152,60	107,00
26	10,10	1	74,00	118,20	109,00
27	10,12	1	46,00	81,20	74,00
28	10,12	1	47,00	71,60	262,00
29	10,14	1	81,00	118,40	63,00
30	10,15	1	53,00	73,00	115,00
31	10,18	1	71,00	137,00	85,00
32	10,19	1	75,00	104,60	67,00
33	10,20	1	62,00	133,80	116,00
34	10,21	1	60,00	114,00	45,00
35	10,22	1	35,00	93,40	43,00
36	10,24	1	33,00	136,60	122,00
37	10,27	1	42,00	105,80	131,00
38	10,28	1	62,00	90,40	58,00
39	10,30	1	45,00	79,20	114,00
40	10,32	1	52,00	77,80	76,00
41	10,32	1	47,00	106,00	70,00
42	10,33	1	81,00	109,60	67,00
43	10,33	1	62,00	67,20	109,00
44	10,35	1	47,00	117,60	47,00
45	10,37	1	45,00	104,80	91,00
46	10,40	1	64,00	88,80	41,00
47	10,41	1	39,00	108,20	99,00
48	10,44	1	65,00	92,40	108,00
49	10,45	1	48,00	139,80	136,00
50	10,45	1	30,00	140,00	180,00
51	10,45	1	71,00	93,40	143,00
52	10,50	1	30,00	107,40	113,00
53	10,51	1	62,00	122,00	220,00
54	10,53	1	57,00	79,20	79,00
55	10,55	1	54,00	91,20	44,00
56	10,57	1	36,00	117,00	125,00
57	10,60	1	41,00	135,40	98,00
58	10,62	1	42,00	160,40	193,00
59	10,64	1	68,00	52,00	35,00
60	10,66	1	43,00	143,40	173,00
61	10,67	1	47,00	138,00	135,00
62	10,68	1	50,00	133,00	45,00
63	10,69	1	32,00	64,00	65,00
64	10,70	1	48,00	90,80	156,00
65	10,72	1	56,00	143,00	80,00
66	10,77	1	64,40	141,60	80,00
67	10,78	1	52,00	116,00	155,00
68	10,78	1	40,00	107,80	161,00
69	10,82	1	61,00	163,60	82,00
70	10,83	1	60,00	73,60	102,00
71	10,89	1	50,00	99,20	199,00
72	10,94	1	47,00	131,40	138,00
73	10,94	1	51,00	97,80	91,00
74	10,95	1	29,00	81,60	112,00
75	10,96	1	50,00	126,80	61,00

76	10,96	1	38,00	111,60	102,00
77	10,96	1	70,00	80,00	135,00
78	10,97	1	61,00	109,60	52,00
79	10,99	1	45,00	94,80	71,00
80	11,00	1	41,00	129,20	114,00
81	11,00	1	61,00	141,40	128,00
82	11,05	1	66,00	129,20	84,00
83	11,06	2	61,00	112,60	177,00
84	11,09	2	42,00	144,60	167,00
85	11,10	2	57,00	96,40	158,00
86	11,10	2	39,00	84,80	191,00
87	11,11	2	64,00	91,20	104,00
88	11,13	2	51,00	82,00	55,00
89	11,13	2	44,00	97,40	73,00
90	11,16	2	46,00	147,60	132,00
91	11,18	2	48,00	151,00	80,00
92	11,19	2	44,00	148,80	156,00
93	11,21	2	30,00	96,20	94,00
94	11,22	2	61,00	116,60	97,00
95	11,22	2	40,00	113,80	141,00
96	11,25	2	38,00	118,00	105,00
97	11,28	2	36,00	100,80	171,00
98	11,30	2	54,00	81,40	158,00
99	11,32	2	55,00	73,80	41,00
100	11,38	2	60,00	127,60	137,00
101	11,42	2	42,00	58,40	173,00
102	11,42	2	64,00	95,00	65,00
103	11,44	2	74,00	88,60	102,00
104	11,46	2	42,00	99,00	190,00
105	11,46	2	73,00	121,40	78,00
106	11,48	2	75,00	110,20	164,00
107	11,49	2	46,00	108,60	242,00
108	11,49	2	51,00	116,20	89,00
109	11,50	2	31,00	79,60	112,00
110	11,51	2	46,00	121,60	137,00
111	11,53	2	52,00	93,60	57,00
112	11,53	2	39,00	146,00	125,00
113	11,54	2	55,00	136,00	145,00
114	11,55	2	42,00	120,60	112,00
115	11,56	2	50,00	87,20	154,00
116	11,58	2	48,00	130,00	90,00
117	11,60	2	63,00	93,80	231,00
118	11,62	2	33,00	138,60	112,00
119	11,62	2	47,00	110,00	180,00
120	11,63	2	36,00	153,60	247,00
121	11,64	2	42,00	89,00	250,00
122	11,64	2	43,00	76,20	139,00
123	11,64	2	55,00	137,40	208,00
124	11,64	2	35,00	100,80	156,00
125	11,67	2	48,00	97,80	46,00
126	11,68	2	46,00	114,40	63,00
127	11,70	2	45,00	99,80	66,00
128	11,72	2	35,00	184,80	121,00
129	11,74	2	59,00	85,20	99,00
130	11,75	2	40,00	77,40	123,00
131	11,76	2	43,00	181,00	145,00
132	11,77	2	51,00	147,60	272,00
133	11,80	2	32,00	58,80	136,00
134	11,83	2	50,00	109,40	253,00
135	11,86	2	46,00	137,40	228,00
136	11,86	2	46,00	67,40	38,00
137	11,87	2	52,00	87,20	244,00
138	11,89	2	52,00	94,80	66,00
139	11,93	2	56,00	105,60	57,00
140	11,94	2	56,00	179,00	155,00
141	11,96	2	51,00	151,60	207,00
142	11,98	2	40,00	69,00	90,00
143	11,99	2	36,00	112,00	130,00
144	12,00	2	45,00	109,80	81,00
145	12,02	2	42,00	162,40	183,00
146	12,04	2	44,00	223,00	120,00
147	12,14	2	31,00	114,40	93,00
148	12,16	2	41,00	138,00	130,00
149	12,17	2	58,00	104,60	182,00
150	12,17	3	45,00	107,20	134,00
151	12,17	2	37,00	118,80	141,00

152	12,21	2	44,00	96,60	82,00
153	12,21	2	52,00	95,00	40,00
154	12,23	2	42,00	182,00	195,00
155	12,24	2	40,00	105,80	396,00
156	12,25	2	44,00	137,00	170,00
157	12,29	2	32,00	90,00	80,00
158	12,31	2	27,00	56,20	254,00
159	12,32	2	50,00	88,40	168,00
160	12,38	2	30,00	160,20	144,00
161	12,41	2	47,00	163,60	107,00
162	12,42	2	65,00	96,80	136,00
163	12,42	2	53,00	72,40	93,00
164	12,43	3	51,00	137,40	193,00
165	12,43	3	66,00	101,80	131,00
166	12,44	3	44,00	147,40	83,00
167	12,45	3	36,00	91,20	114,00
168	12,46	3	40,00	110,80	136,00
169	12,49	3	46,00	98,00	130,00
170	12,51	3	49,00	186,80	61,00
171	12,53	3	49,00	96,80	106,00
172	12,53	3	39,00	159,00	265,00
173	12,57	3	43,00	159,20	259,00
174	12,60	3	41,00	120,80	236,00
175	12,63	3	34,00	97,40	148,00
176	12,64	3	44,00	143,20	94,00
177	12,64	3	56,00	148,60	77,00
178	12,66	3	53,00	108,40	153,00
179	12,70	3	34,00	137,00	120,00
180	12,73	3	40,00	213,20	114,00
181	12,74	3	67,00	80,80	46,00
182	12,74	3	36,00	125,20	99,00
183	12,76	3	32,00	74,60	212,00
184	12,78	3	46,00	144,00	115,00
185	12,81	3	35,00	113,00	75,00
186	12,82	3	59,00	140,80	176,00
187	12,86	3	52,00	128,00	90,00
188	12,87	3	41,00	113,80	91,00
189	12,87	3	52,00	139,00	250,00
190	12,88	3	46,00	152,20	199,00
191	12,89	3	42,00	176,00	105,00
192	12,89	3	35,50	91,90	93,00
193	12,89	3	43,00	77,00	175,00
194	12,92	3	54,00	73,60	122,00
195	12,93	3	42,00	101,40	108,00
196	12,96	3	37,00	106,40	153,00
197	12,96	3	36,00	163,00	275,00
198	12,98	3	46,00	181,60	177,00
199	13,03	3	57,00	96,60	72,00
200	13,04	3	50,00	88,60	207,00
201	13,08	3	37,00	83,80	106,00
202	13,15	3	36,00	201,00	130,00
203	13,16	3	42,00	145,80	126,00
204	13,19	3	39,00	142,40	93,00
205	13,19	3	47,00	104,20	64,00
206	13,19	3	38,00	68,80	101,00
207	13,29	3	40,00	99,20	109,00
208	13,34	3	42,00	130,00	85,00
209	13,36	3	43,00	152,60	172,00
210	13,39	3	38,00	82,00	350,00
211	13,43	3	52,00	128,00	90,00
212	13,55	3	38,00	127,60	232,00
213	13,60	3	60,00	163,80	176,00
214	13,65	3	33,00	88,40	168,00
215	13,66	3	50,00	120,00	80,00
216	13,71	3	31,00	96,20	304,00
217	13,72	3	54,00	157,40	203,00
218	13,73	3	52,00	155,80	291,00
219	13,74	3	46,00	179,40	128,00
220	13,75	3	43,00	126,60	227,00
221	13,76	3	42,00	170,00	115,00
222	13,79	3	56,00	79,60	72,00
223	13,79	3	35,00	147,00	145,00
224	13,82	3	46,00	147,00	150,00
225	13,89	3	37,00	159,40	163,00
226	13,94	3	45,00	119,80	186,00
227	13,98	3	64,00	89,80	71,00

228	13,99	3	42,00	131,60	77,00
229	14,04	3	45,00	159,20	89,00
230	14,31	3	37,00	88,80	121,00
231	14,31	3	39,00	147,20	199,00
232	14,39	3	41,00	122,00	220,00
233	14,43	3	32,00	156,60	172,00
234	14,45	3	49,00	111,20	124,00
235	14,60	3	41,00	96,80	146,00
236	14,61	3	59,00	237,00	210,00
237	14,68	3	53,00	147,60	197,00
238	14,74	3	34,00	85,00	230,00
239	14,90	3	48,00	106,20	214,00
240	14,91	3	50,00	91,40	163,00
241	14,92	3	39,00	147,00	170,00
242	15,02	3	39,00	114,80	146,00
243	15,40	3	42,00	102,40	103,00
244	15,59	3	42,00	158,20	124,00
245	15,65	3	35,00	99,60	252,00
246	16,67	3	69,00	109,40	58,00
