# Supplementary material for: Evidence That Skeletal Muscles Modulate HDL-Cholesterol in Metabolic Healthy Young Adults
Source: Nutrients. 2024 Apr 10;16(8):1110. doi: 10.3390/nu16081110 (PMC11054046; doi:10.3390/nu16081110)
Supplement: Supplementary file 1 [file nutrients-16-01110-s001.zip › Table S4. Regresssion data.pdf]

Outcome: total cholesterol

NORMAL WEIGHT SUBJECTS (n=55)

Characteristic	Beta	95% CI[1]	p-value
Age	2.3	0.60, 4.0	0.009
Sex: male=0, female=1	18	-26, 63	0.405
BMI	3.0	-5.7, 12	0.494
Waist circumference (cm)	-0.02	-2.1, 2.1	0.988
SMI (Kg/m^2)	5.3	-11, 22	0.519

OVERWEIGHT SUBJECTS (n=149)

Characteristic	Beta	95% CI[1]	p-value
Age	1.4	0.58, 2.3	0.001
Sex: male=0, female=1	-20	-42, 2.1	0.076
BMI	0.67	-3.8, 5.2	0.768
Waist circumference (cm)	-0.19	-1.2, 0.78	0.697
SMI (Kg/m^2)	-4.2	-11, 2.6	0.221

Obesi 246 soggetti

Characteristic	Beta	95% CI[1]	p-value
Age	1.6	0.93, 2.3	<0.001
Sex: male=0, female=1	-11	-27, 4.4	0.160
BMI	-0.41	-2.1, 1.3	0.638
Waist circumference (cm)	0.07	-0.54, 0.68	0.827
SMI (Kg/m^2)	0.37	-4.7, 5.5	0.885

Outcome: colesterolo HDL

NORMAL WEIGHT SUBJECTS (n=55)

Characteristic	Beta	95% CI[1]	p-value
Age	0.19	-0.40, 0.77	0.522
Sex: male=0, female=1	15	0.31, 30	0.046
BMI	0.44	-2.5, 3.4	0.767
Waist circumference (cm)	0.18	-0.53, 0.90	0.613
SMI (Kg/m^2)	2.4	-3.1, 8.0	0.388

OVERWEIGHT SUBJECTS (n=149)

Characteristic	Beta	95% CI[1]	p-value
Age	0.06	-0.21, 0.32	0.666
Sex: male=0, female=1	4.5	-2.4, 11	0.196
BMI	-0.15	-1.6, 1.3	0.830
Waist circumference (cm)	-0.09	-0.39, 0.22	0.567
SMI (Kg/m^2)	-1.7	-3.8, 0.41	0.112

PATIENTS WITH OBESITY (n=246)

Characteristic	Beta	95% CI[1]	p-value
Age	0.20	-0.01, 0.40	0.062
Sex: male=0, female=1	2.6	-2.2, 7.4	0.281
BMI	0.25	-0.28, 0.78	0.351
Waist circumference (cm)	-0.14	-0.33, 0.05	0.138
SMI (Kg/m^2)	-1.6	-3.2, -0.03	**0.046**

Outcome: colesterolo LDL

NORMAL WEIGHT SUBJECTS (n=55)

Characteristic	Beta	95% CI[1]	p-value
Age	1.8	0.44, 3.2	0.011
Sex: male=0, female=1	9.2	-26, 45	0.601
BMI	2.2	-4.8, 9.1	0.535
Waist circumference (cm)	-0.22	-1.9, 1.5	0.790
SMI (Kg/m^2)	2.8	-10, 16	0.666

OVERWEIGHT SUBJECTS (n=149)

Characteristic	Beta	95% CI[1]	p-value
Age	1.3	0.54, 2.0	<0.001
Sex: male=0, female=1	-14	-33, 4.6	0.138
BMI	-0.16	-4.0, 3.7	0.933
Waist circumference (cm)	-0.05	-0.87, 0.77	0.905
SMI (Kg/m^2)	-3.0	-8.7, 2.7	0.305

PATIENTS WITH OBESITY (n=246)

Characteristic	Beta	95% CI[1]	p-value
Age	1.3	0.72, 1.9	<0.001
Sex: male=0, female=1	-11	-25, 2.7	0.113
BMI	-0.30	-1.8, 1.2	0.700
Waist circumference (cm)	-0.07	-0.61, 0.48	0.809
SMI (Kg/m^2)	0.58	-4.0, 5.1	0.802

Outcome: Triglycerides

NORMAL WEIGHT SUBJECTS (n=55)

Characteristic	Beta	95% CI[1]	p-value
Age	1.6	-1.0, 4.2	0.226
Sex: male=0, female=1	-31	-97, 36	0.360
BMI	1.9	-11, 15	0.771
Waist circumference (cm)	0.13	-3.0, 3.3	0.935
SMI (Kg/m^2)	0.26	-24, 25	0.983

OVERWEIGHT SUBJECTS (n=149)

Characteristic	Beta	95% CI[1]	p-value
Age	0.56	-1.0, 2.1	0.484
Sex: male=0, female=1	-52	-93, -11	0.014
BMI	4.9	-3.5, 13	0.249
Waist circumference (cm)	-0.26	-2.1, 1.5	0.774
SMI (Kg/m^2)	2.5	-10, 15	0.697

PATIENTS WITH OBESITY (n=246)

Characteristic	Beta	95% CI[1]	p-value
Age	0.44	-0.70, 1.6	0.450
Sex: male=0, female=1	-13	-40, 13	0.332
BMI	-1.8	-4.7, 1.1	0.223
Waist circumference (cm)	1.4	0.34, 2.4	0.009
SMI (Kg/m^2)	7.0	-1.7, 16	0.115
